# Equity should be fundamental to the emergence of innovation

**DOI:** 10.1371/journal.pdig.0000224

**Published:** 2023-04-10

**Authors:** Jack Gallifant, Luis Filipe Nakayama, Judy Wawira Gichoya, Robin Pierce, Leo Anthony Celi

**Affiliations:** 1 Department of Critical Care, Guy’s and St Thomas’ NHS Trust, London, United Kingdom; 2 Laboratory for Computational Physiology, Massachusetts Institute of Technology, Cambridge, Massachusetts, United States of America; 3 Department of Ophthalmology, São Paulo Federal University, Sao Paulo, Sao Paulo, Brazil; 4 Emory University School of Medicine, Department of Radiology, Atlanta, Georgia, United States of America; 5 The Law School, Faculty of Humanities, Arts, and Social Sciences, University of Exeter, Exeter EX4 4HY, United Kingdom; 6 Division of Pulmonary, Critical Care, and Sleep Medicine, Beth Israel Deaconess Medical Center, Boston, Massachusetts, United States of America; 7 Department of Biostatistics, Harvard T.H. Chan School of Public Health, Boston, Massachusetts, United States of America; University of Pittsburgh, UNITED STATES

## Abstract

The ability of artificial intelligence to perpetuate bias at scale is increasingly recognized. Recently, proposals for implementing regulation that safeguards such discrimination have come under pressure due to the potential of such restrictions stifling innovation within the field. In this formal comment, we highlight the potential dangers of such views and explore key examples that define this relationship between health equity and innovation. We propose that health equity is a vital component of healthcare and should not be compromised to expedite the advancement of results for the few at the expense of vulnerable populations. A data-centered future that works for all will require funding bodies to incentivize equity-focused AI, and organizations must be held accountable for the differential impact of such algorithms post-deployment.

## Healthcare systems must support the exploration of new technologies that can improve the overall quality of patient care

Importantly, however, this system must also work fairly for everyone. While ensuring fairness in healthcare delivery is widely considered to be of paramount importance and is rarely disputed, agreeing on what constitutes fair is more complex. Articulating policies that ensure systems deliver in this regard has become increasingly difficult. Whether innovation is fundamental to improving health equity or whether equity should be fundamental to the emergence of innovation has resulted in significant conflict. This is particularly true when deploying algorithms to manage conditions or allocate resources in healthcare settings more efficiently. How stakeholders in the innovation process operationalize recognition of the importance of equity will be crucial to both the viability and trustworthiness of using algorithms in healthcare.

Recent proposals have called for the extension of Section 1557 anti-discrimination requirements to clinical algorithms [1]. The implications are that clinical algorithms would subsequently be regulated as medical devices and other existing laws that seek to penalize the use of biased algorithms in clinical practice. These regulatory measures have generated some resistance [2]. Notably, a recent article expressed concern that applying Section 1557 of the Affordable Care Act, a US law prohibiting treating individuals differently based on protected traits, would stifle innovation and may hold back the medical field [3]. The article objecting to the application of non-discrimination law to algorithms raises four significant concerns: inappropriate extension of anti-discrimination laws, issues related to prospective validation, the inevitability of differential care, and lack of clarity regarding whether and how to prioritize equity and fairness in innovation. Here, we explore these concerns and what they suggest for innovation and healthcare in the future and examine the core relationship between innovation and health equity. Finally, we stress that perspectives critical of the application of anti-discriminatory law to clinical algorithms because it could discourage innovation, must also justify the risk of harm that may be realized from not implementing such policies. While we recognize the complexity of implementing fairness and equity in healthcare and innovation, it is essential to safeguard meaningful commitment to equity.

## Selection decides where innovation occurs

Concerns that greater control over creative operations will stifle discoveries that could otherwise improve the care of the broader population are not new nor unique to healthcare. Indeed, the fear of over-reach of regulation has been held up as a perennial hindrance to beneficial technological advances. However, the selection of what research is permitted to be performed is already in place but controlled mainly by funders, as opposed to regulators.

While regulation may set boundaries, funders decide what areas may be explored. Thus, the knowledge landscape does not advance uniformly but is shaped by both the barriers and incentives of the system in place. Moreover, as is seen in the deployment of artificial intelligence, models reflect the data they are trained upon and can perpetuate biases at scale [4,5]. It is therefore essential to recognize that innovations that emerge from a context where group differences are present can also exacerbate underlying inequities [6].

Therefore, concerns about regulatory oversight of new, potentially discriminatory technologies because they may inhibit innovation are troubling, particularly in the context of persistent health inequities. Thus, failing to enact regulations to address predictable inequitable applications and outcomes could seem irresponsible. The inevitability of the practice of differential care cannot continue to be justified by its longstanding acceptability when it continually and predictably inures to the benefit of the privileged and to the detriment of the already disadvantaged.

Consider the widespread adoption of facial recognition systems in mobile phones despite mass surveillance concerns. Their applications to law enforcement with problematic results have failed to result in a general federal law that regulates facial recognition technology, instead relying on state-by-state laws [7–9]. The Facial Recognition Act, which attempted to establish guidelines and limitations for this technology’s use, was introduced to the Senate in 2019 and failed to be passed in June 2020 [10,11]. In 2023, no current federal laws regulate facial recognition systems. Industry financial incentives drove the innovation of such technology; however, a requirement for such technology to work for all populations before deployment would not have prevented its development. Instead, it would have safeguarded its impact once deployed. Innovation must be encouraged, but its potential value must be calculated based on its impact once applied in the real world, both positive and negative. This final point is often overlooked: the potential harm that could be created through its differential performance after deployment.

Innovators and associated funders often have a financial interest in defeating regulations that limit reach or prevent deployment. This is often in the name of real or perceived benefits for certain groups; however, modern society has acknowledged that financial return cannot come at the cost of harm to others, as this is deemed unfair. Clinical algorithm regulation strikes at the heart of this issue, as we must decide whether fairness means consideration for the effectiveness of the model’s performance on all populations, as opposed to accepting that a model will only work on a limited group. Especially if the group that receives worse performance is already deemed vulnerable. Regulation has a role as the last stopgap to the implementation of biased devices and their systematic re-evaluation. In comparison, funders are the crucial catalyst for equitable innovations. This difference, while subtle, is essential in preventing the dissemination of a message that equity is a barrier to innovation.

## Innovation as an inherently principled enterprise?

The pursuit of new and better technologies, particularly in healthcare, is almost universally supported, yet, innovation, at its core, is essentially indifferent to moral considerations beyond those implicitly imposed by regulation (e.g., safety) or the fortuitous ethical engagement by those involved in bringing a particular innovation to market. It is generally sufficient to show that a new technology works (in some population). However, whether a new health technology underperforms in marginalized populations, or repeatedly disadvantages the already disadvantaged, is currently of no legal or regulatory consequence. Identifying the predictable differential risk of harm due to differentially performing health technologies is a highly appropriate target for regulation, demonstrated by interventions in other sectors. Most notably, the application of non-discrimination laws in housing and employment, domains in which discriminatory treatment has been shown to affect quality of life and lifespan [8].

## Equitable development is not synonymous with equitable outcomes

It should be stressed that regulatory scrutiny of technology design is insufficient to achieve health equity. Consider the management of diabetes, pioneered by the discovery of insulin over a century ago, where it has since saved innumerable patients’ lives [12,13]. In addition, further developments, including self-monitoring devices, intravenous insulin, glucagon, insulin pumps, and better and automated glucose meters, have revolutionized the treatment of diabetes, improving patient outcomes [13]. Despite having an equitable design, Insulin’s impact after distribution has not impacted populations equally. Presently, access to insulin treatment varies significantly, where economic barriers are one of the most significant challenges to treatment access [14,15]. In the USA, health insurance is required to enable insulin access where insurance rates vary across demographics; in sub-Saharan Africa, Oceania, India, and Brazil, patients die due to a lack of regular insulin access [14].

Insulin discovery is an example of health innovation with an equitable design that doesn’t require sex, race, ethnicity, or standing in society optimization but has not equally benefited society—other factors in the market affect outcomes, not just the design process itself. More specifically, the inequities realized are the downstream result of a market-driven healthcare system. Therefore, simply promoting innovation with an equitable design will not lead to the emergence of equity. After these devices have been developed, some form of regulation is required to ensure their impact is fair after entering the market. Setting indications, criteria, or treatment guidelines that are informed by recognition of structural inequalities that could result in the disparate impact of seemingly objective guidelines would significantly complement regulatory oversight of technology design.

## Organizations that develop and deploy algorithms as devices should be accountable for their impact on all groups

The body responsible for overseeing device deployment must ensure that it has created an environment that produces fair results. This can mean designing appropriate incentives or disincentives for each stakeholder in a manner that holds them accountable for their remit—not passing responsibility down the chain. The complexity in the healthcare setting is the significant number of stakeholders involved in care delivery; therefore, it is not always clear who is responsible for such oversight. Ultimately, however, the final responsibility stops with the Government itself, which must delineate these lines of responsibility and bear ultimate responsibility for stewardship. Particularly in the case of health AI deployment, the impact of algorithms on patient subgroups cannot be accurately determined before deployment, where their effects are often only realized afterwards. Further, the impact of an algorithm is dynamic and will change over time, as the groups who are treated vary and the models are updated and recalibrated. While equitable algorithm development is critical, there must be consideration for the model’s continued performance across the lifecycle, to ensure that it remains unbiased, safe, and effective within a changing environment.

Funders should remain responsible for ensuring that the knowledge landscape is advancing health equity and innovators should use an equitable design to maximize the chance of having a fair impact. However, they must iterate and improve as their actual impact is realized at the bedside. Healthcare organizations must play their part, too, and have a responsibility to regularly collect such data and make it available to regulators, researchers, and other required groups. A shift toward the more equitable provision of healthcare requires meaningful change, not only in the way technology is developed but also in its deployment and continued evaluation. Regulators can play a critical role in effecting meaningful change toward health equity. Still, experience has shown that this is only a part of the overall effort needed to achieve equitable healthcare delivery.

Ultimately, the notion that we must disregard equity and fairness to promote innovation must be corrected. The goals of equity and innovation are profoundly compatible. While past strategies may operate as among the many effective paths to innovation, an “equity as optional” approach is unlikely to be the fairest and comes at a cost in terms of health and lives. We believe that fairness demands that value is returned to all patient groups and that accepting less is inappropriate. In practice, no algorithm performs equally on all populations, and innovation will always have differential impacts on groups. Yet, failing to recognize differences in outcomes and the subsequent acceptance of such disparity is unfair. Innovation must have equity at its core, where the goal is to improve outcomes for all groups. This means creating a system where equity and fairness of innovations aim to personalize medicine to individual patients, across all groups; as opposed to broadly performing for some.

The realization of such equity will require a shift to the transparent and continuous evaluation of bias in health outcomes and its reward after subsequent correction. A narrow approach to effecting the change that needs to occur should be expanded beyond regulation to consider alternative ways of supporting the realization of equity as a core component of technological innovation. A rewards system consisting of appropriate incentives could be adopted. Further, systems that reward equity-attentive innovations would require significant collaboration with funding bodies that play a major role in which type of innovation is produced. Rewarding innovations that advance equity may be a more fruitful expedition than punishing the contrary but could, at the very least, serve as an effective complement to regulation, wherein funding bodies will play a significant role in this approach. There must be a fundamental shift in perspective to realize a future of equitable healthcare delivery. Equity should be at the core of health research; innovation should subsequently emerge from this fundamental value; if we envision a data-centered future in healthcare that works for all.
